# Modulation of Interhemispheric Synchronization and Cortical Activity in Healthy Subjects by High-Definition Theta-Burst Electrical Stimulation

**DOI:** 10.1155/2022/3593262

**Published:** 2022-04-29

**Authors:** Van-Truong Nguyen, Chun-Wei Wu, Chien-An Chen, Chao-Chen Lo, Fu-Yu Chen, Chun-I Wu, Pi-Shan Sung, Chou-Ching Lin, Jia-Jin Chen

**Affiliations:** ^1^Department of Biomedical Engineering, National Cheng Kung University, Tainan 701, Taiwan; ^2^School of Biomedical Engineering, College of Biomedical Engineering, Taipei Medical University, Taipei, Taiwan; ^3^Department of Physical Therapy, Shu-Zen Junior College of Medicine and Management, Kaohsiung, Taiwan; ^4^Department of Biomedical Engineering, Chung Yuan Christian University, Taoyuan, Taiwan; ^5^Department of Neurology, National Cheng Kung University Hospital, College of Medicine, National Cheng Kung University, Tainan 701, Taiwan; ^6^Medical Device Innovation Center, National Cheng Kung University, Tainan 701, Taiwan

## Abstract

**Background:**

Various forms of theta-burst stimulation (TBS) such as intermittent TBS (iTBS) and continuous TBS (cTBS) have been introduced as novel facilitation/suppression schemes during repetitive transcranial magnetic stimulation (rTMS), demonstrating a better efficacy than conventional paradigms. Herein, we extended the rTMS-TBS schemes to electrical stimulation of high-definition montage (HD-TBS) and investigated its neural effects on the human brain.

**Methods:**

In a within-subject design, fifteen right-handed healthy adults randomly participated in 10 min and 2 mA HD-TBS sessions: unilateral (Uni)-iTBS, bilateral (Bi)-cTBS/iTBS, and sham stimulation over primary motor cortex regions. A 20-channel near-infrared spectroscopy (NIRS) system was covered on the bilateral prefrontal cortex (PFC), sensory motor cortex (SMC), and parietal lobe (PL) for observing cerebral hemodynamic responses in the resting-state and during fast finger-tapping tasks at pre-, during, and poststimulation. Interhemispheric correlation coefficient (IHCC) and wavelet phase coherence (WPCO) from resting-state NIRS and concentration of oxyhemoglobin during fast finger-tapping tasks were explored to reflect the symmetry between the two hemispheres and cortical activity, respectively.

**Results:**

The IHCC and WPCO of NIRS data in the SMC region under Bi-cTBS/iTBS showed relatively small values at low-frequency bands III (0.06–0.15 Hz) and IV (0.02–0.06), indicating a significant desynchronization in both time and frequency domains. In addition, the SMC activation induced by fast finger-tapping exercise was significantly greater during Uni-iTBS as well as during and post Bi-cTBS/iTBS sessions.

**Conclusions:**

It appears that a 10 min and 2 mA Bi-cTBS/iTBS applied over two hemispheres within the primary motor cortex region could effectively modulate the interhemispheric synchronization and cortical activation in the SMC of healthy subjects. Our study demonstrated that bilateral HD-TBS approaches is an effective noninvasive brain stimulation scheme which could be a novel therapeutic for inducing effects of neuromodulation on various neurological disorders caused by ischemic stroke or traumatic brain injuries.

## 1. Introduction

Noninvasive brain stimulation (NIBS) has been proposed as a potential therapeutic tool to restore and enhance neural function or functional outcome in both psychiatric and neurological disorders [1]. Theta-burst stimulation (TBS), a new modality of NIBS, has been initially introduced as a patterned form of repetitive transcranial magnetic stimulation (rTMS). The TBS protocol consists of a burst of 3 pulses administered at 50 Hz, which is repeated at 5 Hz intervals. TBS can be applied using the intermittent approach (iTBS), in which bursts are delivered for 2 seconds followed by 8 seconds of rest for a train of 10 seconds to induce long-term potentiation-like (LTP) of neuroplasticity [2]. On the other hand, the continuous approach (cTBS) which delivers bursts continuously for a train of 10 seconds to produce long-term depression-like (LTD) of neuroplasticity [3]. Previous studies showed that TBS protocols required lower intensities and shorter durations of stimulation to achieve comparable results [4, 5], but causing longer-lasting effects on motor-evoked potentials and neuronal excitability than conventional rTMS paradigms [6]. To further observe interhemisphere response and effectiveness, bilateral TBS approaches by facilitating on one hemisphere and suppressing on the opposite counterpart have been demonstrated with promising results. A previous study applied 1 Hz rTMS over the right primary motor cortex which facilitated cortical excitability of the left M1 under iTBS [7]. Moreover, a protocol concurrently applying iTBS and cTBS over the left and right of the dorsolateral prefrontal cortex exhibited a reduction of depression rating scale in patients with major depression [8].

Although TMS has beneficial features such as efficacy, safety, and tolerability, the application of TMS is more complicated, costlier, and bulkier and may be associated with high risks of having seizures [9, 10]. In this regard, various forms of TBS used in rTMS have been extended to electrical stimulation schemes to mimic similar brain neuromodulation outcomes [11, 12]. Among various electrical stimulations, the high-definition transcranial direct current stimulation (HD-tDCS), an extension of conventional tDCS, demonstrated a more focused and deeper stimulation like TMS in a safe, well tolerated, and painless way for patients [13]. Investigators showed that a 4 × 1 HD-tDCS configuration with one active electrode placed in the middle and surrounded by four return electrodes could allow electric currents entering deeper and more focal into the human brain cortex [14, 15]. In terms of logistics, HD-tDCS is portable and easy to use via fixed electrode positions over the targeted area using a head cap [9]. However, the effectiveness of HD-tDCS, in particular, and electrical stimulation, in general, is still controversial and has not been approved for clinical treatments like rTMS. Positive outcomes of HD-tDCS were also reported in previous studies, displaying enhancement in cortical excitability [15], functional connectivity [16], and attention [17] in healthy subjects. In contrast, a number of studies revealed that there was no change in both cortical excitatory in healthy subjects [18] and motor performance in stroke patients [19]. Therefore, it is highly desired to develop alternative strategies for improving neuroplasticity and enhancing motor learning.

Earlier studies typically focused on neuromodulatory via direct delivery of electrical current over a single targeted region of the cortex [15, 16, 20]. However, the complexity of functional brain connectivity and the interaction among different brain areas during NIBS might hinder the efficacy of the stimulation. On the contrary, positive results could be obtained by bihemispheric HD-tDCS approaches, which were investigated by simultaneously applying stimulation over two different regions or two hemispheres. Specifically, bilateral HD-tDCS studies indicated significant changes in neurophysiological [21], bimanual sensorimotor performance [22], and the cycling time-trial performance [23]. Therefore, concurrently facilitating cortical regions in the weak hemisphere while suppressing those in the healthy hemisphere might improve the effectiveness of stimulation schemes and benefit for functional brain recovery. In addition, greater improvements in cortical activation in the sensory motor cortex (SMC) region [20] as well as cortical physiology and working memory [24] could be achieved by utilizing concurrent HD-tDCS and motor tasks.

To evaluate the NIBS outcomes, various neuroimaging and electrophysiological measurements have been proposed. Among these, near-infrared spectroscopy (NIRS) is a noninvasive neuroimaging technique which shares similarity to functional magnetic resonance imaging (fMRI) method in terms of monitoring cerebral cortical oxygenation [25]. Moreover, NIRS provides several advantages over fMRI such as portability, affordability, and multichannel measurement. In clinical aspects, NIRS is the optical based technology with high temporal resolution (up to 10 Hz) [26, 27], which is desirable for simultaneously combining with brain electrical stimulation without interference of electrically induced artifacts [28]. In addition to neurophysiological and behavioral outcomes, interhemispheric synchronization and cortical activity within the brain when employing NIBS are important for designing an effective rehabilitation program. Recently, NIRS have been explored to investigate the symmetry between the two hemispheres of the brain in time domain interhemispheric correlation coefficient (IHCC) [29] and time-frequency domain wavelet phase coherence (WPCO) [30], as well as cortical activation [31] in humans. Inspired by these findings and based on our previous studies on the employment of NIRS for evaluating oxygen saturation in skeletal muscle of healthy subjects during exercise [32, 33] and regional brain activity in healthy adults [34, 35] and stroke patients [36], the current study exploits NIRS for the observation of changes in the human brain influenced by NIBS.

In our previous study, we successfully developed an electrical theta-burst stimulator [12], which could provide cTBS and iTBS protocols through two independent channels. Therefore, in the current study, we extended the advantages of TBS protocol through a HD montage (HD-TBS) to investigate its neural effects on human brain function. Our first objective was to examine the influences of unilateral (Uni)-iTBS and bilateral (Bi)-cTBS/iTBS on resting-state interhemispheric and physiological synchronization in different frequency bands. The second objective was to study the effects of these HD-TBS protocols on cortical activation induced by fast finger-tapping exercises. We hypothesized that the Bi-cTBS/iTBS would cause a higher level of asymmetry on resting-state synchronization between the two hemispheres and greater cortical activation than Uni-iTBS and sham treatments. It is therefore expected that this novel combination of electrical TBS protocols with bilateral HD montage could have a potential in extension to poststroke rehabilitation and traumatic brain injuries in the future.

## 2. Methods

### 2.1. Participants

Fifteen healthy persons were recruited to participate in the study (5 females and 10 males, age of 27.5 ± 4.7 years). All participants were right-handed, as assessed with the Edinburgh Handedness Inventory [37]. The subjects had no history of neurological disease, psychiatric disorders, head injuries, or orthopedic issues at upper limb level. Prior to experiments, the investigational procedure, session durations, and possible side effects were announced to the participants. Informed consent was obtained from all subjects involving in the study. This work was performed following the approval of the Institutional Review Board of NCKU Hospital (NCKUH), protocol code: A-BR-109-075.

### 2.2. Experimental Design

**Figure 1:**
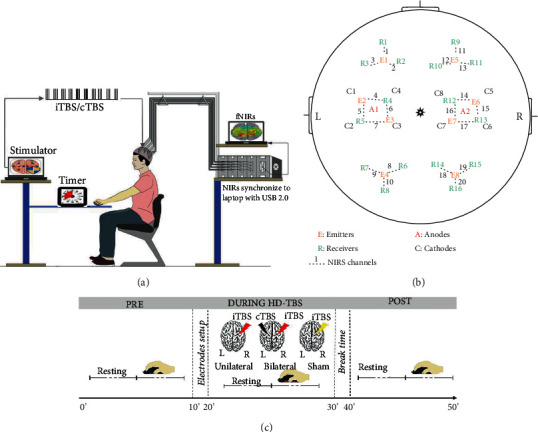
Schematic illustration for experimental design. (a) The experimental setup: subjects sit comfortably on an adjustable chair and worn a 10-20 system electroencephalography (EEG) head cap attached with HD-TBS electrodes and NIRS emitters and receivers; (b) NIRS montage covering bilateral PFC, SMC, and PL and HD-TBS electrodes arrangement; and (c) three phases (pre, during, and post) of each HD-TBS session with resting-state and finger-tapping exercises in each phase.

Subjects randomly participated in three HD-TBS protocols: Uni-iTBS, Bi-cTBS/iTBS, and sham stimulation in a single-blinded design separated by one week to prevent carry-over effects.  Figure 1(a) illustrates the experimental setup. Each stimulation session consists of three phases: (i) pre: before stimulation; (ii) during: during stimulation, and (iii) post: 10 min after stimulation. In each phase, participants began with a 5 min resting-state in sitting position followed by a 5-minute motor task using fast finger-tapping exercise, as shown in Figure 1(c). In the resting-state, participants were asked to maintain resting position with minimal unnecessary movement to avoid interferences to NIRS signals. The resting-state NIRS signals were used to present the interhemispheric synchronization between the two hemispheres. On the other hand, in the motor task condition, all participants performed finger-tapping exercises as fast as possible using their left (nondominant) index and middle fingers to tap alternately on right and left clicks of a wireless computer mouse, which was synchronized to the NIRS system for recording movement rates. The tasks were repeated with ten repetitive blocks of a 10-second motor task followed by a 20-second rest. The start, stop, and number of task blocks were displayed on an LCD monitor, which was placed in front of the subjects for a better control of the tasks. The oxyhemoglobin (HbO) changes in NIRS signals were used to reflect cortical activation induced by the tasks.

### 2.3. HD-TBS Stimulations

Prior to the main experiment, the HD-Explore™ software (Soterix) was used to investigate electric field distribution of a 4 × 1 HD montage in a healthy head model. A 2 mA direct current was set for the anodal electrode (placed over C4 position), while each of the return electrodes (placed over FC6, CP6, CP2, and FC2 in the right hemisphere) was set as -0.5 mA and ~5 cm far from the anodal electrode using the EEG 10-20 system. To understand the potential lead-off situation, one and three of the four cathodes that failed during the simulation were also simulated.

The electrical theta-burst stimulator [12] has been adopted in the current study to provide monophasic TBS-like (cTBS/iTBS) protocols. For safety issues, the stimulator was powered by a 5 V direct current source and a 5 s ramp up and ramp down set at the beginning and ending of each session to avoid discomfort for subjects. The 4 × 1 HD montage was arranged using five small ring electrodes made by AgCl with a diameter of 12 mm for each hemisphere. Two anode electrodes (A1 and A2) were placed in the center of stimulation circles over C3 and C4 positions of EEG 10-20 system and surrounded by four cathode electrodes (at a distance of ~5 cm from the active electrode), which were, respectively, located on FC5, CP5, CP1, and FC1 and FC6, CP6, CP2, and FC2 for left and right hemispheres (Figure 1(b)). In both Uni-iTBS and Bi-cTBS/iTBS sessions, a 2 mA (~17.69 A/m^2^) current was delivered to the stimulation region in a 10 min period (5-second ramp up, 9 minutes and 50 seconds at 2 mA, and 5-second ramp down). For the Uni-iTBS session, the iTBS protocol was delivered to the right primary motor cortex. We selected the iTBS instead of cTBS for unilateral stimulation due to its potentiation in rehabilitation by promoting LTP-like effects. For the Bi-cTBS/iTBS session, both cTBS and iTBS protocols were simultaneously delivered to the left and right primary motor cortex. In the sham condition, the iTBS protocol was applied to the right hemisphere with only a 15-second activation at the beginning and ending of the session (5-second ramp up, 5 seconds at 2 mA, and 5-second ramp down), no current pulses in the middle duration of 9 minutes 30 seconds.

### 2.4. NIRS Recording and Data Analysis

#### 2.4.1. NIRS Recording of Hemodynamic Response

**Figure 2:**
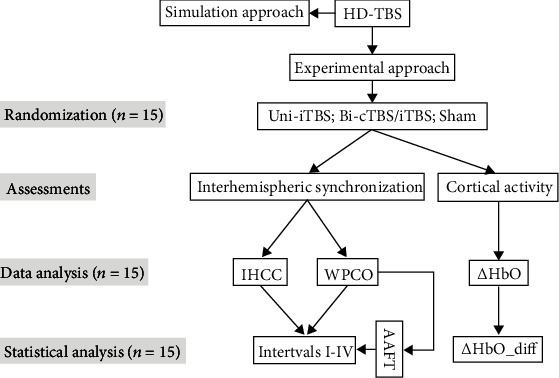
Data analysis flowchart.

The commercially available continuous wave NIRS system model NIRScout1624 (NIRx Medical Technologies Berlin, Germany) was utilized to measure hemodynamic changes in resting-state and during fast finger-tapping exercise by HD-TBS stimulations. The NIRS measurement montage was designed using eight infrared light emitters with two light-emitting diodes with wavelengths of 760 and 850 nm and sixteen receivers (with a 3 cm interdistance between each emitter and receiver), accordingly providing a total of twenty NIRS channels with a sampling measurement rate of 7.8 Hz [38]. Figure 1(b) shows the position of NIRS emitters (E1-E8 placed over F3, CFC5, CCP3, P3, F4, CFC6, CCP4, and P4, respectively) and receivers (R1-R16 placed over FAF5, F1, FFC5, CFC3, CCP5, P1, PCP5, PPO5, FAF6, F2, FFC6, CFC4, CCP6, P2, PCP6, and PPO6, respectively) based on EEG 10-20 system. The mapping of optodes covered the left and right PFC (channels 1-3 and 11-13), SMC (channels 4-7 and 14-17), and parietal lobe (PL) (channels 8-10 and 18-20), respectively. The system could detect changes in the levels of HbO, deoxyhemoglobin (HbR), and total hemoglobin (HbT) in the cortex. Furthermore, the HbO oscillation and HbO concentration of all channels within one area were averaged to present the interhemispheric synchronization in resting-state and cortical activation induced by fast finger-tapping exercise.  Figure 2 shows the data analysis flowchart of the current study.

#### 2.4.2. NIRS Data Preprocessing

The nirsLAB package was used to compute optical density and thus concentration of oxyhemoglobin, which was proposed to be most sensitive to the changes of brain physiological signals [31]. In addition, we analyzed the power spectrum of each time series data after detrending for determination of NIRS signal quality. A detection of peak values around 1 Hz reflects cardiac pulsation in NIRS signals, indicating a good contact between optical probes and scalp [39]. All NIRS channels without appearance of cardiac pulsation were excluded from data analysis. MATLAB software (MathWorks Inc., MA, USA) was used for all offline data analyses.

#### 2.4.3. Resting-State NIRS Data Analysis

A 3rd-order Butterworth band-pass filtered with a cutoff frequency range of 0.02–2 Hz was applied to eliminate slow drift motion artifacts and unwanted high-frequency noises. In addition, the isolated signals were further categorized into four interested frequency bands: I (0.7–2 Hz); II (0.15–0.7 Hz); III (0.06–0.15 Hz); and IV (0.02–0.06 Hz), indicating responses of cardiac, respiratory, myogenic, and neuronal activity, respectively [40].

The zero-lag cross correlation between a pair of NIRS channels on two hemispheres was denoted as IHCC, which presented the interhemispheric hemodynamic synchronization in time domain [29, 36]. IHCCs from each pair of NIRS channels were calculated from signal band-pass filtered according to four frequency bands I-IV and then averaged to reflect the symmetry of the two hemispheres within a cortical region at pre, during, and post HD-TBS stimulations. IHCC's value ranges between -1 and 1, implying desynchronization and perfect synchronization between the two hemispheres within a region [29, 41]. WPCO, introduced in previous studies, was based on continuous wavelet transform and provided information on instantaneous phase shift of two signals in a time-frequency domain [30, 42, 43]. WPCO from each pair of NIRS channels in four frequency bands I–IV were calculated using sine and cosine information of two HbO signals and then averaged to display the phase symmetry of the two hemispheres. The WPCOs were measured within a cortical region at pre, during, and post HD-TBS stimulations. WPCO's value ranges between 0 and 1. A WPCO value equaling 1 indicates well synchronization of the two hemispheres, while that equaling 0 signifies desynchronization between regions of the two hemispheres at the particular frequency. In addition, a 100 surrogate signals of the amplitude adjusted Fourier transform (AAFT) was adopted to investigate statistical significance of WPCO [44]. It was considered to be statistically significant when WPCO of two HbO signals equals or higher than WPCO average of 100-surrogate data plus two times of standard deviation [42]. Fisher's *Z*-transform was applied for non-Gaussian distribution of IHCC and WPCO before statistical analysis.

#### 2.4.4. NIRS Data during Motor Task Analysis

The changes of HbO concentration (△HbO) were utilized as a biomarker for cortical activation during fast finger-tapping exercises [31]. Firstly, a 3rd-order Butterworth band-pass filtered with a cutoff frequency range of 0.005–0.2 Hz was applied for eliminating possible slow drift motion artifacts and high-frequency components from respiratory rate and cardiac pulsation. We defined △HbO as the mean of HbO concentration changes in 15 seconds with 10 seconds during the task and 5 seconds after the end of the task in each block due to delayed hemodynamic responses to the task [45]. Secondly, △HbO from block 2 to block 10 of all NIRS channels within a cortical region were averaged to obtain block average response in each cortical regions. The △HbO from block 1 was removed from data analysis because it might delay in response. Finally, a difference of △HbO (△HbO_diff) between the right (targeted) and left (control or conditioning) hemispheres within a cortical region were calculated to reveal cortical activation during fast finger-tapping exercise at the pre, during, and post HD-TBS stimulations.

### 2.5. Statistical Analysis

SPSS version 25.0 was used in this study for statistical analysis. Two-way repeated measures ANOVA analysis was employed to investigate interaction effect by stimulation session (Uni-iTBS, Bi-cTBS/iTBS, and sham) as the first within-subject factor and phase (pre, during, and post) as the second within-subject factor on transformed IHCC, transformed WPCO, and △HbO_diff. One-way repeated measure ANOVA was performed when there was significant interaction by stimulation session and phase to test a relative time changes in each HD-TBS session as well as among the three stimulation sessions in the same phase. The Bonferroni was used in post hoc test for multiple comparisons. A *p* value ≤ 0.05 was considered statistically significant in all the tests.

## 3. Results

### 3.1. Simulation of Electric Field Distribution for the 4 × 1 HD Montage

**Figure 3:**
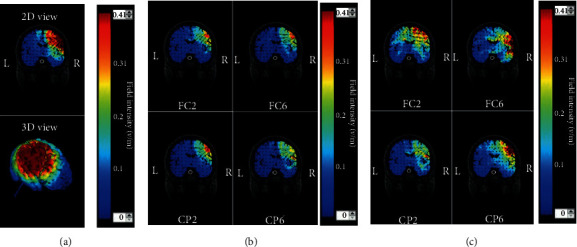
The electric field distribution for the 4 × 1 HD montage generated by HD-Explore™. (a) All four cathodes were valid, (b) one cathode failed, and (c) only one cathode was valid.

Figure 3 illustrates electric field distribution for the 4 × 1 HD montage, with 2 mA for the anode and -0.5 mA for each of the four cathodes, generated by the HD-Explore™ software (Soterix).  Figure 3(a) shows a focalized electric field distribution in both 2D and 3D view when all four cathodes were valid during simulation with maximal field intensity of 0.348 (V/m). The focality of the electric field distribution was maintained when one of the four cathodes (FC2, CP2, CP6, or FC6) failed with field intensity, respectively, changed to 0.362 V/m, 0.410 V/m, 0.327 V/m, and 0.348 V/m, as shown in Figure 3(b). However, the loss of spatial focality was observed, and the maximal field intensity was significantly enhanced to 0.348 V/m, 0.550 V/m, 0.690 V/m, and 0.470 V/m for a valid cathode placing over FC2, CP2, CP6, or FC6, as separately shown in Figure 3(c).

### 3.2. Effects of HD-TBS on Resting-State Interhemispheric Synchronization

#### 3.2.1. IHCC in Different Frequency Bands

**Figure 4:**
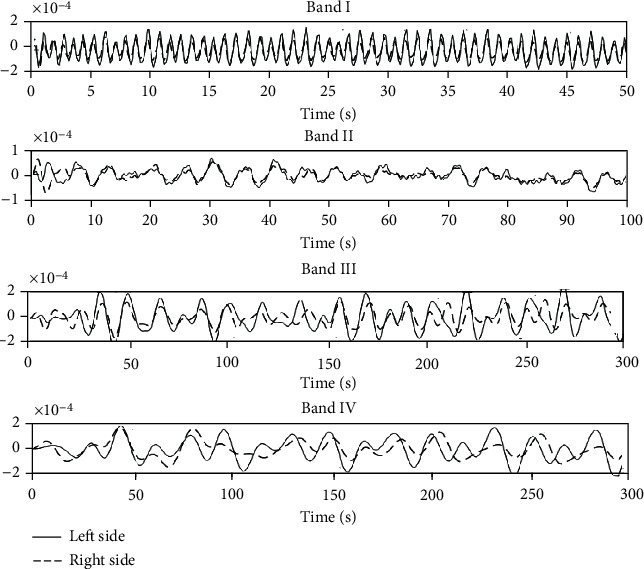
Representative resting-state IHCCs in the frequency bands I–IV in the SMC during Bi-cTBS/iTBS stimulation. (a–d) IHCCs from band I to IV, respectively. Solid line (–) and dashed line (--) indicate HbO oscillation in the left and right hemispheres, respectively.

Figure 4 shows representative IHCCs in the four frequency bands I–IV in the SMC during Bi-cTBS/iTBS stimulation. Compared to the high IHCCs in frequency bands I and II, relatively low values of 0.62 and 0.48 can be found in the bands III and IV, respectively.

**Figure 5:**
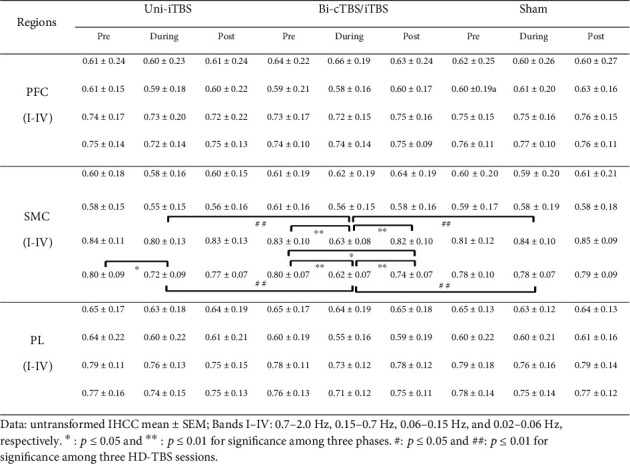
IHCCs in different cortical regions under three HD-TBS protocols. Data: untransformed IHCC mean ± SEM; bands I–IV: 0.7–2.0 Hz, 0.15–0.7 Hz, 0.06–0.15 Hz, and 0.02–0.06 Hz, respectively. ^∗^*p* ≤ 0.05 and ^∗∗^*p* ≤ 0.01 for significance among three phases. ^#^*p* ≤ 0.05 and ^##^*p* ≤ 0.01 for significance among three HD-TBS sessions.

The untransformed IHCCs in all frequency bands I–IV in the pre, during, and post phases of HD-TBS sessions are shown in Figure 5. Repeated measure two-way ANOVA showed that there was a significant interaction by stimulation session and phase on transformed IHCCs in the bands III and IV in the SMC (*F* (4, 56) = 14.494, *p* < 0.01, *ƞ*^2^ = 0.509 and *F* (4, 56) = 5.677, *p* < 0.01, *ƞ*^2^ = 0.289, respectively). The Bonferroni post hoc tests for different phases comparisons indicated that the IHCC in the band III in the SMC during Bi-cTBS/iTBS (0.63 ± 0.08) was significantly lower than that in the pre- (0.83 ± 0.10, *p* < 0.01) and poststimulation (0.82 ± 0.10, *p* < 0.01). In group comparison, the IHCC during Bi-cTBS/iTBS was significantly lower than those during Uni-iTBS (0.80 ± 0.13, *p* < 0.01) and sham (0.84 ± 0.10, *p* < 0.01). In band IV, the comparison among different phases in the SMC showed that the IHCC during Uni-iTBS (0.72 ± 0.09) was significantly lower than that in the prestimulation (0.80 ± 0.09, *p* < 0.05). The IHCC during Bi-cTBS/iTBS (0.62 ± 0.07) was significantly lower than those in the pre- (0.80 ± 0.07, *p* < 0.01) and poststimulation (0.74 ± 0.07, *p* < 0.01), respectively. In addition, the IHCC in post Bi-cTBS/iTBS was significantly lower than that in the prestimulation. In group comparison, the IHCC during Bi-cTBS/iTBS was significantly lower than those in the Uni-iTBS (0.72 ± 0.09, *p* < 0.05) and sham (0.78 ± 0.07, *p* < 0.01). The results suggest that the interhemispheric synchronization in time domain in low-frequency bands III and IV could be remarkably affected by the 10 min and 2 mA Bi-cTBS/iTBS stimulation. There were no obvious changes in the IHCCs in the bands I and II in the SMC region as well as bands I–IV in the PFC and PL areas for all conditions.

#### 3.2.2. WPCO in Different Frequency Bands

**Figure 6:**
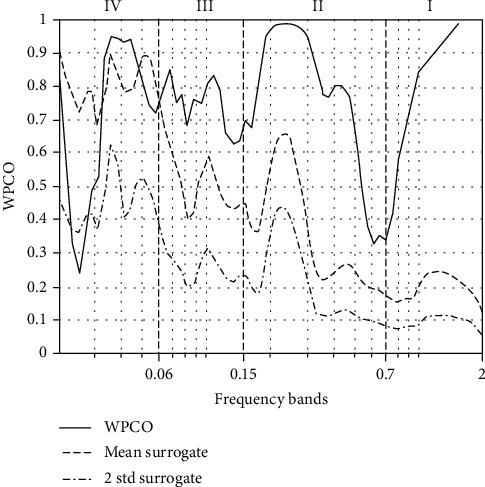
Representative resting-state WPCOs in the bands I–IV in the SMC by Bi-cTBS/iTBS stimulation. The solid line (-) indicates WPCOs of the bands I–V; the dashed and dotted line (-.) shows WPCO of 100 AAFT; the dashed line (--) displays two standard deviations of 100 WPCO of AAFT; vertical dashed lines reveals different divisions of the frequency bands.

Figure 6 shows a typical WPCO measured in the SMC region during Bi-cTBS/iTBS stimulation with relatively low values in bands III and IV. The WPCOs at the bands I–V (solid line) were greater than those of 100 AAFT, and their two times of standard deviation (dashed line) indicated a statistical significance of WPCO.

**Figure 7:**
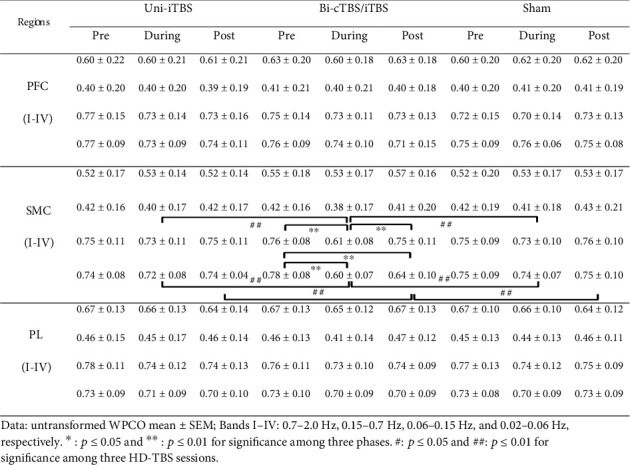
WPCOs in different cortical regions under three HD-TBS protocols. Data: untransformed WPCO mean ± SEM; bands I–IV: 0.7–2.0 Hz, 0.15–0.7 Hz, 0.06–0.15 Hz, and 0.02–0.06 Hz, respectively. ^∗^*p* ≤ 0.05 and ^∗∗^*p* ≤ 0.01 for significance among three phases. ^#^*p* ≤ 0.05 and ^##^*p* ≤ 0.01 for significance among three HD-TBS sessions.

The untransformed WPCO values in all frequency bands in the pre-, during, and poststimulations under three HD-TBS sessions are shown in Figure 7. Repeated measure two-way ANOVA suggested that there was a significant interaction between stimulation session (Uni-iTBS, Bi-cTBS/iTBS, and sham) and phase (pre, during, and post) on transformed WPCOs in the bands III and IV in the SMC (*F* (4, 56) = 12.399, *p* < 0.01, *ƞ*^2^ = 0.470 and *F* (4, 56) = 13.287, *p* < 0.01, *ƞ*^2^ = 0.487, respectively). The Bonferroni post hoc tests implied that WPCOs in the band III in the SMC during Bi-cTBS/iTBS (0.61 ± 0.08) was significantly lower than those in the pre- (0.76 ± 0.08, *p* < 0.01) and poststimulations (0.75 ± 0.11, *p* < 0.01), as well as during Uni-iTBS (0.73 ± 0.11, *p* < 0.01) and sham (0.73 ± 0.10, *p* < 0.01). Additionally, WPCOs in the band IV in the SMC region during and post Bi-cTBS/iTBS (0.60 ± 0.07 and 0.64 ± 0.10) were significantly lower than that in the prestimulation (0.78 ± 0.08) with *p* value < 0.01 for both comparisons. Furthermore, WPCOs in the band IV in the SMC during and post Bi-cTBS/iTBS were significantly lower than those in the Uni-iTBS (0.72 ± 0.08, *p* < 0.01, and 0.74 ± 0.04, *p* < 0.01) and sham (0.74 ± 0.07, *p* < 0.01, and 0.75 ± 0.10, *p* < 0.01), respectively. Our results reveal that Bi-cTBS/iTBS has a considerable reduction in phase synchronization in low-frequency bands. There were no substantial changes in WPCOs of the bands I and II in the SMC region as well as bands I–IV in the PFC and PL areas for all conditions.

### 3.3. Finger-Tapping Exercise Induced Cortical Activation under HD-TBS Stimulations

**Figure 8:**
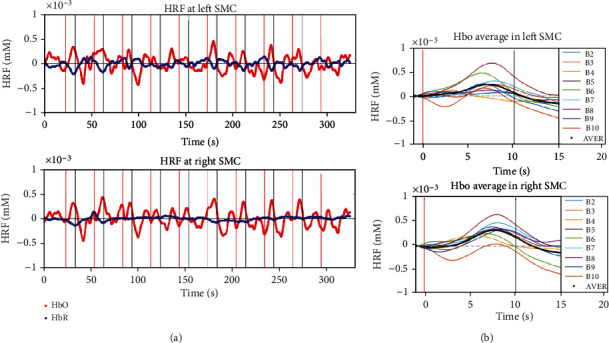
(a) Typical hemodynamic responses in the left and right SMC induced by fast finger-tapping exercises and Bi-cTBS/iTBS stimulation. Red and blue plots indicate the fluctuation of HbO and HbR, respectively; vertical lines indicate the start and stop of the tasks. (b) Block average HbO concentration (black bold plots) from block 2 to block 10.

The analysis of finger-tapping movement rate indicated that there was no notable interaction effect between session and phase (F (4, 56) = 1.98, *p* = 0.19, *ƞ*^2^ = 0.14). There was also no noteworthy difference in the main effect for session (*p* = 0.11) and phase (*p* = 0.80). The results demonstrated that movement rates were not significantly different to influence the analysis of cortical activation in all conditions.  Figure 8(a) illustrates the hemodynamic response function (HRF) in the left and right SMC regions during concurrent Bi-cTBS/iTBS and fast finger-tapping exercises. The red plots indicate a strong response of HbO to the tasks, while the blue graphs show a slight change of HbR fluctuation.  Figure 8(b) presents the HbO average (black bold plots) with higher amplitudes in the right SMC as compared to the left counterpart.

**Figure 9:**
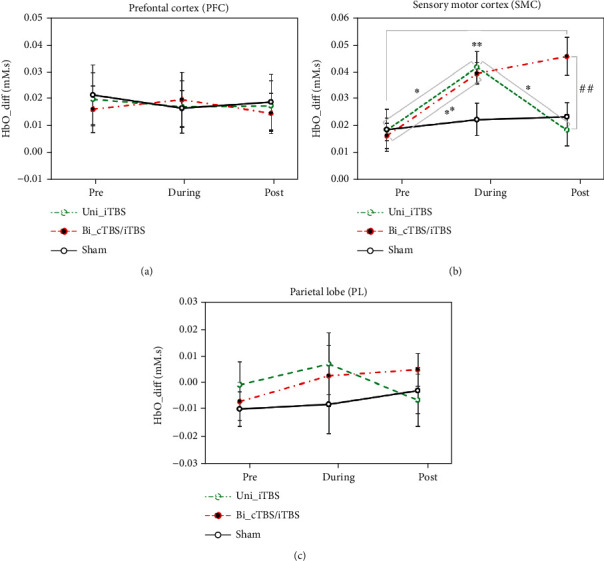
Group average of △HbO_diff (±SEM) in the (a) PFC, (b) SMC, and (c) PL measured in the pre, during, and 10 min poststimulations of the three HD-TBS sessions. ^∗^*p* ≤ 0.05 and ^∗∗^*p* ≤ 0.01 indicate a significant difference among three phases of each HD-TBS session. ^#^*p* ≤ 0.05 and ^##^*p* ≤ 0.01 indicate a significant difference among three HD-TBS sessions in the same phase.

The ANOVA analysis revealed a significant interaction effect between session and phase in the SMC for △HbO_diff (*F* (4, 56) = 4.821, *p* < 0.01, *ƞ*^2^ = 0.256).  Figure 9(b) shows the results of the Bonferroni post hoc tests indicating that △HbO_diff during Uni-iTBS (0.045 ± 0.006 mM) was remarkably greater than that in the prestimulation (0.022 ± 0.008 mM) with *p* < 0.05 and poststimulation (0.022 ± 0.006 mM) with *p* < 0.05. In addition, △HbO_diff during Bi-cTBS/iTBS (0.043 ± 0.004 mM) and poststimulation (0.049 ± 0.007 mM) was greatly higher than that in the prestimulation (0.020 ± 0.005 mM) with *p* value < 0.01 for both comparisons. Furthermore, △HbO_diff in the post Bi-cTBS/iTBS was significantly larger than that in the Uni-iTBS with *p*-value < 0.01. The results imply that the SMC cortical activation could be strongly induced by fast finger-tapping exercise, which was applied during Uni-iTBS as well as during and poststimulation of Bi-cTBS/iTBS. In contrast, cortical activation in the PFC and PL, as, respectively, shown in Figures 9(a) and 9(c), were not significantly different in all conditions.

## 4. Discussion

In the current study, three electrical HD-TBSs with an intensity of 2 mA for 10 minutes were used to modulate the brain of healthy persons. The stimulation characteristics are as common as those reported in earlier investigations of electrical brain stimulation [46]. As reported by the participants, twelve out of fifteen subjects realized the appearance of theta-burst pulse at the beginning of each session and the sensations lasted after about thirty seconds. Thus, the present work demonstrated the feasibility and safety of the TBS protocols as evidenced by no severe adverse events among all recruited participants.

The focality of electric field distribution was examined by both simulation and experimental approaches. In the simulation, our results showed that the 4 × 1 HD montage could generate a localized current distribution if three or four cathodes were valid (Figures 3(a) and 3(b)), demonstrating a good agreement with earlier findings using a computational model [38]. In contrast, the loss of focality could be observed when only one cathode was valid during stimulation (Figure 3(c)). However, the limitation of the simulation approach is that only one side stimulation could be tested. In addition to simulation on focality, the SMC was within the stimulation region, but the PFC and PL were outside of stimulation ranges in our NIRS experiment design. Observed from the results of our resting-state experiment, the interhemispheric hemodynamic synchronization (IHCC and WPCO) was significantly decreased in the SMC region during Bi-cTBS/iTBS protocol but not in the PFC and PL regions which reflected the focality of stimulation current with a HD montage. These findings coincide with the focality of HD-tDCS reported elsewhere [15]. Our extension of TBS protocols with bilateral HD approaches therefore may have benefits for the future development of bilateral stimulation for poststroke rehabilitation.

### 4.1. Effects of HD-TBS on Resting-State Hemodynamic Synchronization

In line with our hypothesis, there was a degree of interhemispheric desynchronization in the low-frequency bands under the three HD-TBS sessions. The bihemispheric SMC physiological oscillations in the bands III (0.06–0.15 Hz) and IV (0.02–0.06 Hz) were significantly desynchronized by Bi-cTBS/iTBS stimulation. It is known that the band III (0.06–0.15 Hz) reflects myogenic activity of smooth muscle cells [40]. The myogenic mechanism responds to variations within intravascular pressure and is mostly active in the small vessels of the brain. On the other hand, band IV (0.02–0.06 Hz) presents neurogenic activity [40]. Our results are highly correlated with the previous studies on the effects of HD-tDCS on low-frequency physiological signals in the brain [16, 47]. The IHCC derived from HbO of NIRS signals was proposed to reflect the strength and direction of linear relationship between the two hemispheres in time domain [29, 36]. However, functional brain connectivity presented by single time-domain analyses may be misleading due to the fact that physiological signals from the brain are not always linear. Therefore, to enhance the accuracy of the NIRS assessment, WPCO was employed to quantify the relationship between two sets of data based on phase difference in time-frequency domain [48–50]. In contrast to low-frequency bands, we observed no significant changes in IHCCs and WPCOs in the bands I (0.7–2 Hz) and II (0.15–0.7 Hz), indicating the contribution of cardiac and respiratory activities to blood flow of the brain [40].

Interestingly, we only observed the desynchronization in the SMC region following the Bi-cTBS/iTBS stimulation, but not Uni-iTBS and sham stimulations. It is worth noting that research exploring effects of bilateral NIBS on cortical oscillation still remain limited. The homeostatic mechanism of the brain is a function that maintains overall synaptic excitation at a stable level [51]. The homeostasis mechanism might be a reason for the unchanged interhemispheric synchronization under the Uni-iTBS stimulation. However, in the Bi-cTBS/iTBS, 2 mA currents were simultaneously delivered to the left and right primary motor cortex. In this bihemispheric stimulation, there were 4 mA difference in total between two sides of the SMC region, hence probably enhancing the threshold of homeostatic and causing a remarkable change in low-frequency physiological oscillations. The current study supports the previous findings on the importance of bilateral stimulation on both cortical excitability [7, 21] and behavioral performance [22, 23].

Overall, the mechanism of interhemispheric stimulation has not been fully explored. A previous investigation focused more on a single hemisphere stimulation and proposed that the theta frequency of TBS protocol could modulate high-frequency gamma rhythms, resulting in cortical excitability [52]. It has been hypothesized that iTBS can cause LTP to result in facilitation of cortical activity by increasing the amplitude of late I-waves [2]. In contrast, cTBS reduces the excitability of late I-waves and provides long-term depression (LTD) in excitatory synaptic connection [3]. Our findings showed that only Bi-cTBS/iTBS stimulation could lower the resting-state interhemispheric synchronization in the low-frequency bands. It implies the combined effects of concurrent cTBS and iTBS for suppressing the left hemisphere while facilitating the right hemisphere. These findings might be a crucial point for the treatment of unilateral ischemic stroke towards enhancing the activity of the affected hemisphere while reducing the activity of the unaffected one.

### 4.2. Effects of Concurrent HD-TBS and Finger-Tapping Exercise on Cortical Activation

We also examined the effects of HD-TBS and fast finger-tapping exercise on cortical activation. The △HbO is commonly used as a biomarker to reflect cortical activation during a motor task [31]. In this study, instead of using purely △HbO in the targeted (right) hemisphere, the △HbO difference (△HbO_diff) between right and left hemispheres within a region was used to reveal cortical activation induced by concurrent HD-TBS and finger-tapping exercises. Previous studies proposed that cTBS protocol causes inhibition and iTBS protocol causes excitation of cortical excitability [2, 3], and another study demonstrated that both hemispheres are involved in skill movement [53]. By using △HbO_diff to reflect cortical activation during finger-tapping task and HD-TBS sessions, the interaction between ipsilateral and contralateral hemispheres were both considered. Thus, the effects of external factors such as different times between phases and sessions could be minimized. Our results suggested that the SMC activation was considerably enhanced when the finger-tapping exercise was applied simultaneously with both Uni-iTBS and Bi-cTBS/iTBS sessions. In contrast, cortical activation in the PFC and PL was unchanged in all conditions. Our findings coincide with the findings of the previous studies indicating that during the motor tasks, the SMC region was engaged showing the most evident changes [31, 54]. However, finger-tapping movement rate was comparable in all conditions, implying that acute effects of HD-TBS only cause influences at metabolic level but behavioral level.

Cortical activation during a motor task is proposed to be under control of neurovascular coupling function, in which cortical regions that related to the task would be supplied with more blood than other regions to maintain adequate oxygen and glucose for neuron activity [55]. With NIRS assessment, the cortical activation induced by fast finger-tapping exercise was able to be observed at before, during, and after HD-TBS stimulations with minimal electric interference. Our results showed that cortical activation, reflected by △HbO_diff during Uni-iTBS and Bi-cTBS/iTBS, was significantly greater than that in the prestimulations. These findings confirmed the efficacy of electrical HD-TBS interventions in inducing cortical activation when applied simultaneously with motor tasks. Our results are in line with the previous studies, proposing that when there is a motor activity during the prolonged electrical brain stimulation involving the same brain areas, the amount of current that enters the SMC triggers further changes in brain activity patterns [20, 56].

Generally, the SMC cortical activation during fast finger-tapping exercise was significantly enhanced during Uni-iTBS and Bi-cTBS/iTBS sessions; however, the phenomenon was only maintained until 10 min poststimulation in the bilateral approach. It is known that homeostasis maintains the threshold of physiological response in the brain in a safe range for protecting man's brain [51]. The longer-lasting effect is a phenomenon which has been proposed for treatment of TBS [6]. In the Uni-iTBS session, a 2 mA iTBS might cause LTP-like in the targeted hemisphere (right) of the brain, resulting in the enhanced cortical activation. However, in the Bi-cTBS/iTBS session, both cTBS and iTBS are concurrently applied to the left and right primary motor cortex, by which the SMC region was modulated by a double of 2 mA difference between the two hemispheres. The approach of Bi-cTBS/iTBS might cause a greater threshold of homeostatic and lead to a higher asymmetry of cortical activity during fast finger-tapping exercise and provide longer-lasting effects. Our findings agree well with the previous studies using bilateral montage of conventional tDCS and HD-tDCS, consequently inducing longer-lasting effects on motor performance and sensory motor activation [22, 57].

### 4.3. Study Limitations

There were several limitations to the current study. Firstly, the sample size was small, and only healthy adults were recruited to participate in the study. Further study should recruit more subjects with a broader range of ages (younger and older) as well as patients with neurological disease such as unilateral ischemic stroke or traumatic brain injuries to confirm the initial findings of the present work. Secondly, we did not include conventional HD-tDCS or HD-cTBS stimulations as comparison groups in our study. Thirdly, the current study was single-blind crossover design; the experimenter did know the sequences of HD-TBS sessions. This could potentially have caused bias when conducting the sessions.

## 5. Conclusion

This study highlights the effects of different HD-TBS stimulation protocols on resting-state interhemispheric synchronization and cortical activation induced by fast finger-tapping exercise in healthy adults. We firstly confirmed the focality of the 4 × 1 HD montage by both simulation and experimental methods. NIRS study of both IHCC and WPCO showed that the interhemispheric synchronization in the SMC region at low-frequency bands was generally desynchronized during and post Bi-cTBS/iTBS stimulation in comparison to those in the prestimulation, Uni-iTBS, and sham sessions. This implies that bilateral stimulation approaches are more effective in modulating the functional connectivity in the resting-state. In addition, the SMC activation during Uni-iTBS session was significantly higher than those in the pre- and poststimulation, suggesting that the bilateral approaches of HD-TBS may cause longer-lasting effects after stimulation. Similar observations could be found in Bi-cTBS/iTBS, displaying significant enhancement in both during and 10 min poststimulation sessions. Our observation from NIRS measurements of interhemispheric synchronization and cortical activation signifies the effectiveness of the bilateral stimulation protocol of HD-TBS. Future studies could extend the experiment and assessment methods presented in the current work to neurological and psychiatric disease populations such as traumatic brain injuries or poststroke rehabilitation.

## Data Availability

The demographic data and data of outcome assessment used to support the findings of this study are available from the corresponding author upon request.
